# Mechanism of emergency phytoremediation technology based on a 3D-QSAR pharmacological model

**DOI:** 10.3389/fpls.2024.1324144

**Published:** 2024-08-26

**Authors:** Minghao Li, Siming Wang, Shimei Sun

**Affiliations:** ^1^ School of Emergency Science and Engineering, Jilin Jianzhu University, Changchun, China; ^2^ XingYe Environmental Group Co., Ltd., Harbin, China

**Keywords:** sudden environmental pollution accidents, emergency phytoremediation techniques, plant resistance, phytodegradability, molecular dynamics, quantitative molecular surface analysis

## Abstract

**Introduction:**

The ability of transgenic plants to respond to sudden environmental pollution accidents has become viable. Nonetheless, there is a dearth of research regarding the mechanism by which transgenic plants degrade organic pollutants. Hence, this study aimed to elucidate the process of organic pollutant degradation by plants, offering theoretical support for the application of transgenic plant emergency phytoremediation technology.

**Methods:**

In this investigation, we developed a 3D-QSAR pharmacophore model to represent the collective impact of plant resistance and phytodegradation. This was achieved by employing integrated effect values following treatment with a sine function approach. Moreover, we have undertaken an inaugural exploration of the coregulatory mechanism involved in plant resistance and pollutant degradation within plants. Additionally, we applied virtual molecular modification techniques for analysis and validation, striving for a more indepth understanding of the molecular-level enhancement mechanism related to the degradation of pollutants within plant organisms.

**Results and discussion:**

The mechanism analysis results of the Hypo 1 pharmacophore model were verified, indicating that hydrophobic characteristics affect the resistance and degradation of PCBs in plants, significantly affecting the degradation effect of pollutants in plants.

## 1 Introduction

With the rapid advancement of industrialization, human social activities have significantly expanded, leading to a surge in the frequency of sudden environmental pollution accidents (SEPAs). These incidents pose a substantial threat to human development and ecological security due to their high uncertainty, intricate evolution, rapid propagation, and severe wide-ranging damage (Lei et al., 2018; Wang and Wang, 2021). In light of the increase in environmental pollution, finding a method capable of promptly eliminating pollutants at their source and addressing pollution issues arising from SEPA holds vital practical significance for global ecological wellbeing and sustainable human development.

The primary techniques for addressing environmental pollution include physical, chemical, biological, and integrated remediation methods (Li and Sun, 2022). Among these, phytoremediation, as a biological approach, offers the advantages of simplicity and cost-effectiveness compared to traditional methods, with the added benefit of minimizing secondary pollution. However, traditional phytoremediation suffers from protracted remediation cycles (Terzaghi et al., 2019). With the maturation of transgenic technology, scientists have begun enhancing phytoremediation efficiency from a transgenic standpoint to overcome its lengthy remediation time. For instance, Kong et al. (2022) introduced the N-demethylase gene into soybeans, resulting in the removal of 98% and 84% of isoproturon from water and soil within 5 and 14 days, respectively, in transgenic soybeans. This achievement outpaced that of conventional soybeans, underscoring the efficacy of transgenic plants in organic matter remediation (Kong et al., 2022). The researchers incorporated two bacterial genes designed to degrade RDX, a military explosive residue, into *Panicum virgatum*, demonstrating its ability to remove and degrade RDX at a rate of 27 kg per hectare (Cary et al., 2021). These studies illustrate the potential of transgenic plants for addressing SEPA, yet there is limited research on the mechanism of pollutant degradation within phytoremediation technology. Therefore, this paper aims to investigate the mechanism of organic pollutant degradation by plants, providing a theoretical foundation for the use of transgenic plants in emergency remediation technology.

The activity of the plant antioxidant system closely correlates with the development of stress tolerance. During plant growth, various abiotic stresses can increase intracellular reactive oxygen species concentrations, leading to oxidative stress and plant damage (Gechev et al., 2006). In response to oxidative damage, plants have evolved various antioxidant mechanisms, including peroxidase (POD), which significantly influences plant stress resistance (Kawano, 2003). When organic pollutants infiltrate plants, they can induce oxidation in cell membranes and various organelles, inhibiting POD enzyme activity and compromising plant health (Zhang et al., 2017). Additionally, during the degradation of organic pollutants by plants, plants can secrete bioxygenases related to organic pollutant degradation, affecting the overall degradation efficiency (Mohammadi et al., 2007). Among these pollutants, polychlorinated biphenyls (PCBs) are among the 12 persistent organic pollutants (POPs) regulated under the Stockholm Convention due to their environmental persistence, high biotoxicity, substantial bioconcentration, and long-range transport characteristics (Hayat et al., 2019; Terzaghi et al., 2020). Nevertheless, PCBs continue to leach into the soil environment as by-products of incinerating chlorinated compounds or producing and using chlorine-containing chemicals, potentially causing sudden environmental incidents. In summary, the enzymatic structures of peroxidase (1CCK), an enzyme related to plant resistance, and dioxygenase (3GZX), an enzyme related to plant degradability, were derived from the Protein Data Bank (http://www1.rcsb.org), and these enzymes were utilized as target enzymes to jointly analyze phytoremediation techniques using PCB molecules as representatives of the degradation mechanism.

3D-QSAR models integrate structural information of molecules in three dimensions with their biological activities, enabling a more accurate and comprehensive understanding of the underlying mechanisms involved in phytoremediation. Given the complexity and diversity of POPs, there is a pressing need for the development of advanced 3D-QSAR models tailored specifically for phytoremediation. These models could not only help researchers select the most promising plant species for remediation but also guide the design of novel virtual modifications to pollutants that could improve their degradability by plants. In this paper, we established a 3D-QSAR pharmacophore model for predicting the plant resistance and phytodegradability of PCBs based on their molecular structural parameters. We selected PCBs with documented plant resistance and phytodegradability data as dependent variables and employed molecular structural parameters as independent variables to construct the model. Furthermore, we validated the model by incorporating molecular virtual modification techniques, molecular dynamics methods, and quantitative molecular surface analysis. Based on these results, additionally, it provides a theoretical reference for the genetic modification of highly efficient repair plants in the future.

## 2 Materials and methods

### 2.1 Characterization of plant resistance receptor enzymes and plant degradative enzymes that bind to PCBs—molecular docking

From the PDB database, we selected a representative plant resistance receptor enzyme (PDB ID: 1CCK) and a plant degradation receptor enzyme (PDB ID: 3GZX) as acceptors, along with 60 PCBs as ligands. These proteins were loaded into Discovery Studio 2020 software for analysis. The LibDock module was employed to investigate the interaction between the receptor enzymes and the ligand molecules (Zhang et al., 2019). Using the LibDock module, we defined the two selected enzymes as two enzyme receptors. Subsequently, molecular docking was conducted for each of the 60 PCB ligands with these two enzyme receptors using the “Find Sites from Receptor Cavities” option under the Define module to identify potential binding sites within the receptors. Docking preferences were configured as custom, Max Hit to Save was set to 10, and all other settings remained at their default values. The resulting scoring function values *X*_i,1_ and *X*_i,2_ (*i* = 1, 2), the principal parameters of the LibDock module, were separately computed to better characterize the interaction between the two plant receptor enzymes and the 60 PCB ligands, and the results are presented in **Table 1**.

**Table 1:** Calculated values for the combined effect of plant resistance and degradability for 60 PCBs.

PCBs (IUPAC)	Plant resistance	Phytodegradability	Comprehensive effect value
1	75.426	71.577	1.865
2	81.624	73.685	1.91
4	73.822	76.604	1.867
6	83.766	78.019	1.929
7	83.786	74.232	1.923
8	87.006	76.220	1.943
9	80.980	71.669	1.900
10	72.398	77.082	1.857
11	87.533	74.788	1.943
12	88.323	78.408	1.952
13	88.073	75.084	1.946
14	87.991	73.595	1.942
16	69.318	79.249	1.836
17	83.794	77.495	1.929
18	71.252	70.436	1.831
20	79.668	76.524	1.905
23	82.007	71.477	1.905
25	89.403	74.420	1.950
28	89.893	75.407	1.954
31	86.392	67.811	1.913
33	90.196	75.792	1.956
36	91.012	68.505	1.936
39	90.431	71.216	1.944
40	73.154	80.691	1.865
46	65.282	78.958	1.803
52	67.751	77.703	1.822
59	79.861	74.233	1.901
61	93.144	76.448	1.968
67	92.454	60.891	1.896
74	85.467	70.231	1.919
77	99.318	73.871	1.98
81	99.705	75.858	1.985
85	64.546	70.043	1.775
90	75.506	62.585	1.820
95	70.561	78.021	1.844
101	71.570	65.686	1.811
105	99.271	65.437	1.945
110	71.019	56.458	1.740
114	98.329	66.982	1.951
118	85.897	72.296	1.928
123	95.620	70.652	1.960
126	105.165	69.209	1.973
128	66.586	64.601	1.765
130	72.689	72.932	1.850
135	72.564	74.071	1.852
138	69.900	58.690	1.750
153	67.731	55.717	1.708
156	89.360	51.817	1.803
157	88.695	51.693	1.799
164	73.038	48.786	1.677
167	89.597	55.942	1.844
169	109.961	49.844	1.825
174	67.737	43.346	1.571
180	72.489	44.462	1.622
189	84.203	53.788	1.799
196	74.020	44.223	1.629
200	76.580	53.269	1.749
201	70.293	47.688	1.644
207	76.815	56.916	1.785
209	79.575	53.317	1.769

### 2.2 Comprehensive effect values for plant resistance and degradability of PCBs corrected by the sine normalization method

The scoring function is normalized using Equation 1 as follows:


(1) $$X_{i,1}=\frac{xi,1}{x1max};X_{i,2}=\frac{xi,2}{x2max}\;\left(\text{i}=1,\;2,\;3,\ldots ,23\right)$$

Following the normalization of Equation 1, a simple sine function was chosen to further normalize the data. A lower score after docking with plant resistance enzymes indicates a lower toxicity of PCBs to plants. Conversely, higher SFs after docking with plant degradation enzymes suggest that PCBs are less resistant to degradation by these enzymes, thus enhancing their environmental remediation potential. To ensure consistency in the direction of change for both effects, a negative sign was introduced before the scoring function of PCBs and plant resistance enzymes. This produced a combined activity value reflecting the synergy of the two effects in an inverse direction. The sine method (Wang et al., 2017; Julien et al., 2007; Liu and Marukawa, 2004) generated a combined score *k_i_* (*i* = 1, 2, 3, 4,…,60), representing the impact of PCBs on both types of plant receptor enzymes, as shown in Equation 2:


(2) $$k_{i}=1-sin(\frac{\pi X_{i,\;1}}{2})+sin(\frac{\pi X_{i,\;2}}{2})\;\left(i=1,\;2\right)$$

The calculated values of the combined effect of resistance and degradability of the PCB plants after sine normalization treatment are shown in **Table 1**.

### 2.3 Pharmacophore modeling of the comprehensive effects of plant resistance and PCB degradability

A “pharmacophore” represents the molecular structure of a wide range of active features common to PCB congeners (Langer, 2010). These pharmacophore elements can include hydrophobic groups, specific chemical groups, etc (Huang et al., 2014; Feng et al., 2016). The development of 3D-QSAR pharmacophore models helps reveal the relationship between PCB activity and structural characteristics, aiding mechanistic analysis (Kubo et al., 2003). We employed the combined effect of PCB resistance and degradation as the dependent variable and PCB structure as the independent variable. The 3D-QSAR Pharmacophore Generation module within Discovery Studio 2020 was utilized to construct the 3D-QSAR model for the combined effect of PCB resistance and degradation. This module allowed us to investigate the mechanisms influencing the combined effect of PCB resistance and degradation.

The selected 3D-QSAR pharmacophore features included hydrogen bond acceptors, hydrogen bond donors, hydrophobic groups, hydrophobic–aliphatic groups, and aromatic rings. The module parameters were set as follows: Fast for conformation generation, an energy threshold of 20 kcal/mol, maximum conformations per homolog set to the default 255, and minimum interference distance set to 0. The energy threshold for similarity conformation generation was set to 10 (Zhao et al., 2021; Zhang et al., 2021).

The HypoGen module within Discovery Studio 2020 software was chosen to evaluate the constructed 3D-QSAR pharmacophore model for the combined effects of plant resistance and degradation of PCBs. The cost function, a key metric, reflects the model’s suitability and includes its complexity, chemical properties, and error between predicted and experimental data (Chen and Li, 2021). According to Occam’s Razor, the optimal pharmacophore model should have the lowest total cost among all models and be close to the fixed cost value (Vincent and Bellini, 2002). Another significant parameter is the configuration cost, which is determined by the model’s spatial complexity. A meaningful pharmacophore model should possess a configuration cost value of no more than 17 (Arooj et al., 2011).

### 2.4 Molecular dynamics methods

The magnitude of the binding of plant resistance receptor enzymes (1CCK) and plant degrading enzymes (3GZX) to PCBs indicates the strength of the interaction between contaminants and enzymes (Gu et al., 2020). In this study, we performed molecular dynamics simulations using Gromacs software for PCB molecules and enzymes before and after modification utilizing the Dell PowerEdge R7425 server. Each complex was placed in a periodic dodeca cube with a side length of 10 nm. Molecular confinement was conducted using the GROMOS96 43a1 force field, and Na^+^ ions were added to neutralize the system charge. The PCB–enzyme complex system underwent energy minimization simulations via the steepest gradient method, with 50,000 simulation steps. The simulation duration for both the hot and pressure baths was set to 100 ps, with the pressure bath size maintained at a constant standard atmospheric pressure of 1 bar. Kinetic simulations lasting 200 ps were performed for each step group to calculate the changes in binding energy between PCBs and enzymes. Finally, the binding energy data generated via the molecular dynamics method were combined with quantitative molecular surface analysis to analyze the phytoremediation capacity.

### 2.5 Quantitative molecular surface analysis

Interactions between proteins and ligands are typically governed by weak interactions among biomolecules (Xie et al., 2006). Common weak interactions include van der Waals forces, hydrogen bonding forces, and hydrophobic forces (Johnson et al., 2010). In this study, we utilized the quantitative molecular surface analysis function of Multiwfn software (Lu and Chen, 2012a, b) to calculate the van der Waals surface area before and after molecular modification, with the electron density of the equivalent surface set to 0.002 a.u. We analyzed the reasons for changes in van der Waals forces resulting from alterations in indicators before and after PCB molecular modification. Subsequently, we investigated the factors influencing changes in the binding energy between plant resistance receptor enzymes and plant-degrading enzymes and PCBs.

## 3 Results and discussion

### 3.1 Construction and evaluation of a 3D-QSAR pharmacophore model for the comprehensive effects of plant resistance and degradability of PCBs

The program generated a total of nine 3D-QSAR pharmacophore models for the combined effect of plant resistance and degradation of PCBs, as outlined in **Table 2**.

**Table 2:** Nine pharmacophore models and their statistical data.

Hypo No. 1	Total cost	RMS	Correlation	Features
1	216.167	0.05	0.6	H, RA*2
2	216.065	0	0	HA, RA*2
3	216.173	0.06	0.5	H, RA*2
4	216.065	0	0	H, RA*2
5	216.178	0.06	0.5	RA*2
6	216.065	0	0	RA*2
7	216.182	0.06	0.48	H*2, RA
8	216.065	0	0	H*4
9	216.888	0.16	−0.01	HA, H, RA
Configuration	13.12	Fixed cost	201.816	
Null cost	227.037			

HBA, hydrogen bond acceptor; HBD, hydrogen bond donor; H, hydrophobic group; HA, hydrophobic aliphatic group; RA, aromatic ring.

Based on the parameter values of the nine 3D-QSAR pharmacophore models (**Table 2**), five valid models (Hypo 1, Hypo 3, Hypo 5, Hypo 7, and Hypo 9) were selected because their RMS (Root mean square) and correlation values were both greater than zero. Among the five effective 3D-QSAR pharmacophore models, Hypo 1 exhibited the most promising performance. Its Total cost value (216.167) was closest to the Fixed cost value (201.816) and the Nullcost value (227.037). The RMS value (0.05) and Total cost value (216.167) were also the smallest among the five models. These parameters collectively indicated that Hypo 1 outperformed the others in representing the combined pharmacophore characteristics of plant resistance and degradation of PCBs. The configuration parameter, which should be less than 17 for a pharmacophore model to be considered significant, was 13.12 for the Hypo 1 model, confirming its significance. **Table 3** shows that the error values of the predicted values from the Hypo 1 pharmacophore model for the combined effect values of the 60 PCBs were less than 2, which falls within an acceptable error range. This outcome further validated the reliability of the Hypo 1 pharmacophore model and its ability to represent the combined pharmacophore characteristics of plant resistance and degradation of PCBs.

**Table 3:** Statistical data of 60 PCBs based on Hypo 1.

PCBs (IUPAC)	Fit value	Est value	Act value	Error
1	3.881	1.061	1.104	−1.040
2	3.896	1.026	1.072	−1.045
4	3.858	1.117	1.127	−1.009
5	3.870	1.089	1.087	1.002
7	3.865	1.101	1.061	1.038
8	3.867	1.096	1.049	1.045
9	3.883	1.055	1.069	−1.013
10	3.864	1.102	1.138	−1.032
11	3.898	1.021	1.044	−1.023
12	3.922	0.966	1.046	−1.083
13	3.899	1.017	1.043	−1.025
14	3.919	0.972	1.039	−1.069
16	3.870	1.089	1.163	−1.068
17	3.873	1.081	1.067	1.013
18	3.870	1.088	1.129	−1.038
20	3.871	1.085	1.089	−1.004
23	3.865	1.100	1.063	1.035
25	3.888	1.043	1.035	1.007
28	3.867	1.096	1.036	1.058
31	3.886	1.047	1.025	1.022
33	3.892	1.034	1.035	−1.001
36	3.898	1.021	1.008	1.013
39	3.895	1.028	1.022	1.006
40	3.866	1.099	1.135	−1.033
46	3.867	1.095	1.196	−1.093
52	3.872	1.084	1.175	−1.084
59	3.870	1.089	1.083	1.005
61	3.896	1.025	1.025	−1.000
67	3.889	1.041	0.958	1.086
74	3.883	1.057	1.040	1.016
77	3.922	0.965	1.003	−1.040
81	3.952	0.902	1.006	−1.116
85	3.901	1.012	1.182	−1.168
90	3.873	1.082	1.057	1.023
95	3.877	1.071	1.153	−1.077
101	3.874	1.077	1.104	−1.025
105	3.901	1.013	0.968	1.046
110	3.872	1.083	1.042	1.040
114	3.923	0.963	0.978	−1.016
118	3.920	0.971	1.045	−1.077
123	3.912	0.988	1.002	−1.014
126	3.953	0.899	0.977	−1.087
128	3.879	1.067	1.137	−1.066
130	3.899	1.017	1.127	−1.109
135	3.875	1.075	1.131	−1.052
138	3.869	1.091	1.069	1.021
153	3.869	1.092	1.061	1.029
156	3.938	0.930	0.889	1.046
157	3.887	1.047	0.891	1.175
164	3.878	1.067	0.949	1.125
167	3.931	0.945	0.928	1.018
169	3.953	0.898	0.825	1.088
174	3.933	0.941	0.924	1.019
180	3.884	1.054	0.901	1.170
189	3.917	0.976	0.933	1.046
190	3.880	1.063	1.013	1.050
200	3.899	1.017	0.972	1.046
201	3.884	1.053	0.957	1.101
207	3.882	1.058	1.005	1.053
209	3.880	1.063	0.954	1.114

### 3.2 Mechanistic analysis of the effect of PCBs on plant resistance and degradation based on the characteristic map of the 3D-QSAR pharmacophore model

**Figure 1:**
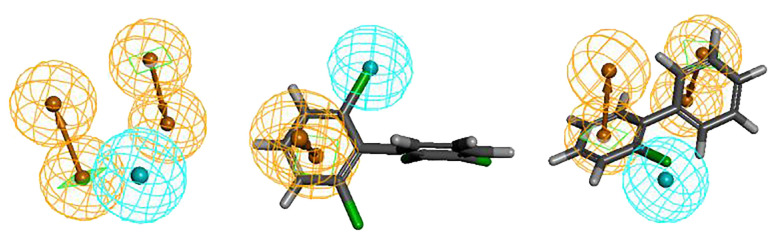
Spatial relationship of Hypo1 and alignment with PCBs, PCB-1 and PCB-46.

The Hypo 1 pharmacophore model comprised one hydrophobic and two aromatic ring features. **Figure 1** shows an overlay of the Hypo 1 pharmacophore model with the three-dimensional spatial relationships of PCBs, where blue represents hydrophobic features and yellow represents aromatic ring features. Using PCB-46, which had the highest combined effect value, and PCB-1, which had the lowest number of chlorine atoms, as examples, the positions of the hydrophobic and aromatic ring features were visualized (**Figure 1**). Although the number of molecules with varying aromatic ring features differed slightly, all the molecules contained these two features. This indicated that these two features had a significant impact on the combined effect of plant resistance and degradation of PCBs. Since the aromatic ring is an inherent property of PCB structures, modifying the hydrophobic features of PCBs in plants can further mitigate their resistance and enhance their phytodegradability.

Furthermore, the aromatic ring feature reflects the susceptibility of PCBs to electrophilic substitution reactions, where they tend to lose electrons in this region. Previous studies have shown that the E_HOMO_ parameter, which measures the electron loss ability (reducibility) of PCBs, can influence plant resistance to some extent (Li et al., 2021). Therefore, the aromatic ring indirectly reflects the E_HOMO_ parameter of PCBs (electron loss property or reducibility). Hydrophobic features represent hydrophobic functions contained in aliphatic atoms. The literature indicates that the primary influential parameter for plant degradation of PCBs *in vivo* is Log*P*, which measures the hydrophobicity of organic compounds (Li et al., 2021). Hydrophobic features partially reflect the influence of Log*P* parameters on PCB plant degradability. In summary, the 3D-QSAR pharmacophore model for the combined effect of plant resistance and degradation of PCBs somewhat corroborates the literature findings (Li et al., 2021). However, further analysis is needed to understand how the hydrophobic characteristics of pollutants indirectly affect PCB resistance in plants.**Figure 1** shows that the hydrophobic feature significantly influences PCB plant resistance and degradability and is located at the 2′ position of the benzene ring, indicating that the hydrophobic group at this position can affect PCB plant resistance and degradability. Therefore, this chapter validates the mechanism of the effect of hydrophobic features on PCB plant resistance and degradability using molecular virtual modification techniques.

### 3.3 Validation and investigation of the mechanism analysis results based on molecular virtual modification technology

#### 3.3.1 Validation of the mechanism analysis results using molecular virtual modification techniques

**Figure 2:**
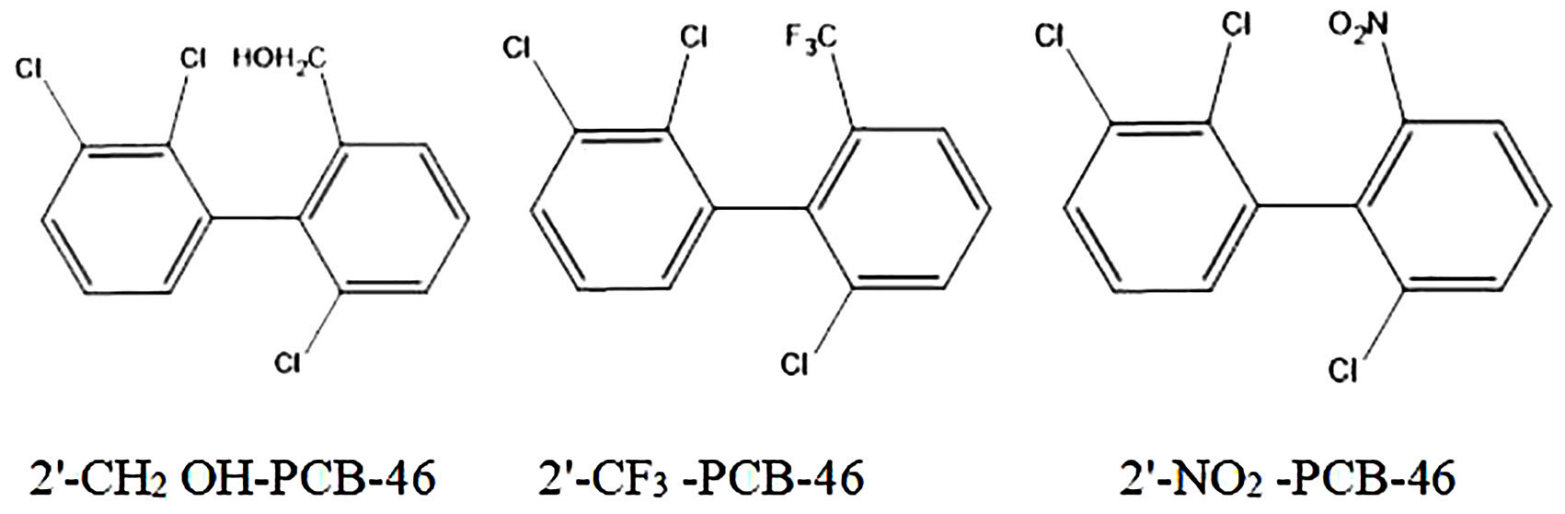
2D structure diagram of three PCB-46 virtual modification molecules.

We selected three common hydrophobic groups as substituents: hydroxymethylol (-CH_2_OH), trifluoromethyl (-CF_3_), and nitryl (-NO_2_). PCB-46, which had the greatest comprehensive effect, served as the template molecule for molecular virtual modification to adjust PCB resistance and degradation. We conducted molecular virtual modification to adjust PCB resistance and degradation using PCB-46 as the template molecule. The Hypo 1 pharmacophore model was used to predict the combined effects of three molecules (2′-CH_2_OH-PCB-46, 2′-CF_3_-PCB-46, and 2′-NO_2_-PCB-46) after virtual modification of PCB-46 (**Figure 2**).

**Table 4** presents the changes in the combined effect values of plant resistance tolerance and degradability before and after PCB-46 modification. The combined effects of all three PCB-46 virtual modification molecules (2′-CH_2_OH-PCB-46, 2′-CF_3_-PCB-46, and 2′-NO_2_-PCB-46) tended to increase, with changes of 10.03%, 50.92%, and 97.66%, respectively. These changes indicated significant alterations in plant resistance and degradability after introducing hydrophobic feature group modifications to PCBs, confirming the results from the mechanistic analysis of the Hypo 1 pharmacophore model, which indicated that hydrophobic features significantly affected PCB resistance and degradability changes.

**Table 4:** Changes in the comprehensive effect before and after virtual modification of PCB-46.

No.	PCBs	Comprehensive effect value	Change (%)
1	PCB-46	1.196	
2	2′-CH_2_ OH-PCB-46	1.316	10.03
3	2′-CF_3_ -PCB-46	1.805	50.92
4	2′-NO_2_ -PCB-46	2.364	97.66

Using the 2D-QSAR model of plant resistance and plant degradability of PCBs constructed in previous literature (Li et al., 2021), we further predicted and analyzed the plant resistance and plant degradability data for the three molecules after virtual modification of PCB-46 (**Table 5**). The results revealed that 2′-CH_2_OH-PCB-46 exhibited improved plant stress resistance and plant degradability, consistent with the trend of the combined effect values. Hence, we employed 2′-CH_2_OH-PCB-46 as an illustrative case study to conduct an in-depth examination of plant stress resistance and plant degradability alterations before and after virtual modification with PCBs. Thus, we selected 2′-CH_2_OH-PCB-46 as a representative example to scrutinize the changes in plant stress resistance and enhancement in phytodegradability mechanisms before and after PCB modification.

**Table 5:** Changes in plant resistance and phytodegradability before and after PCB-46 modification.

Molecular	Plant resistance	Range of variation (%)	Phytodegradability	Range of variation (%)
PCB-46	65.2815		78.9577	
2′-CH_2_OH-PCB-46	41.1463	−36.97	87.0085	10.20
2′-CF_3_-PCB-46	10.3862	−84.09	59.4146	−24.75
2′-NO_2_-PCB-46	115.2215	76.50	90.5838	14.72

Based on the results from the 2D-QSAR model analysis of PCB phytodegradability [31], reducing hydrophobic interactions and increasing hydrophilic interactions between PCBs and phytodegradable enzyme binding facilitated PCB degradation. An analysis of the phytodegradability improvement mechanism of 2′-CH_2_OH-PCB-46 revealed that although the hydrophobic -CH_2_OH group was introduced, the hydrophobicity of 2′-CH_2_OH-PCB-46 decreased to 4.81 after this addition, showing a slight decrease compared to the hydrophobicity of PCB-46 (4.83). This decrease improved the affinity of 2′-CH_2_OH-PCB-46 for plant degradation enzymes, leading to enhanced plant degradability of the molecule. Similarly, in comparison to that of PCB-46, the hydrophobicity of 2′-NO_2_-PCB-46 (4.68) decreased, while that of 2′-CF_3_-PCB-46 (6.30) increased, resulting in opposite trends in the phytodegradability changes for both, with 2′-NO_2_-PCB-46 and 2′-CH_2_OH-PCB-46 demonstrating positive trends in phytodegradability improvement.

#### 3.3.2 Analysis of the mechanism using molecular docking and molecular dynamics

Reactions between enzymes and ligands are typically governed by intermolecular forces (Li et al., 2022). Common weak interaction forces include van der Waals forces, hydrogen bonding forces, and hydrophobic forces, among others (Johnson et al., 2010). To analyze the mechanism of plant resistance improvement by PCBs, the “Show 2D Diagram” function in the “View Interaction” module of Discovery Studio 2020 software was used to extract the active pockets of PCB-46 and 2′-CH_2_OH-PCB-46 bound to plant resistance enzymes. This allowed us to analyze the virtual modification of PCB-46 before and after the changes in weak forces within the active pocket. Green represents interactions involving van der Waals forces between amino acid residues and the molecule, light pink indicates electrostatic forces, purple signifies π–π stacking, red represents unfavorable donor–donor interactions, and yellow signifies π–cation interactions.

**Figure 3:**
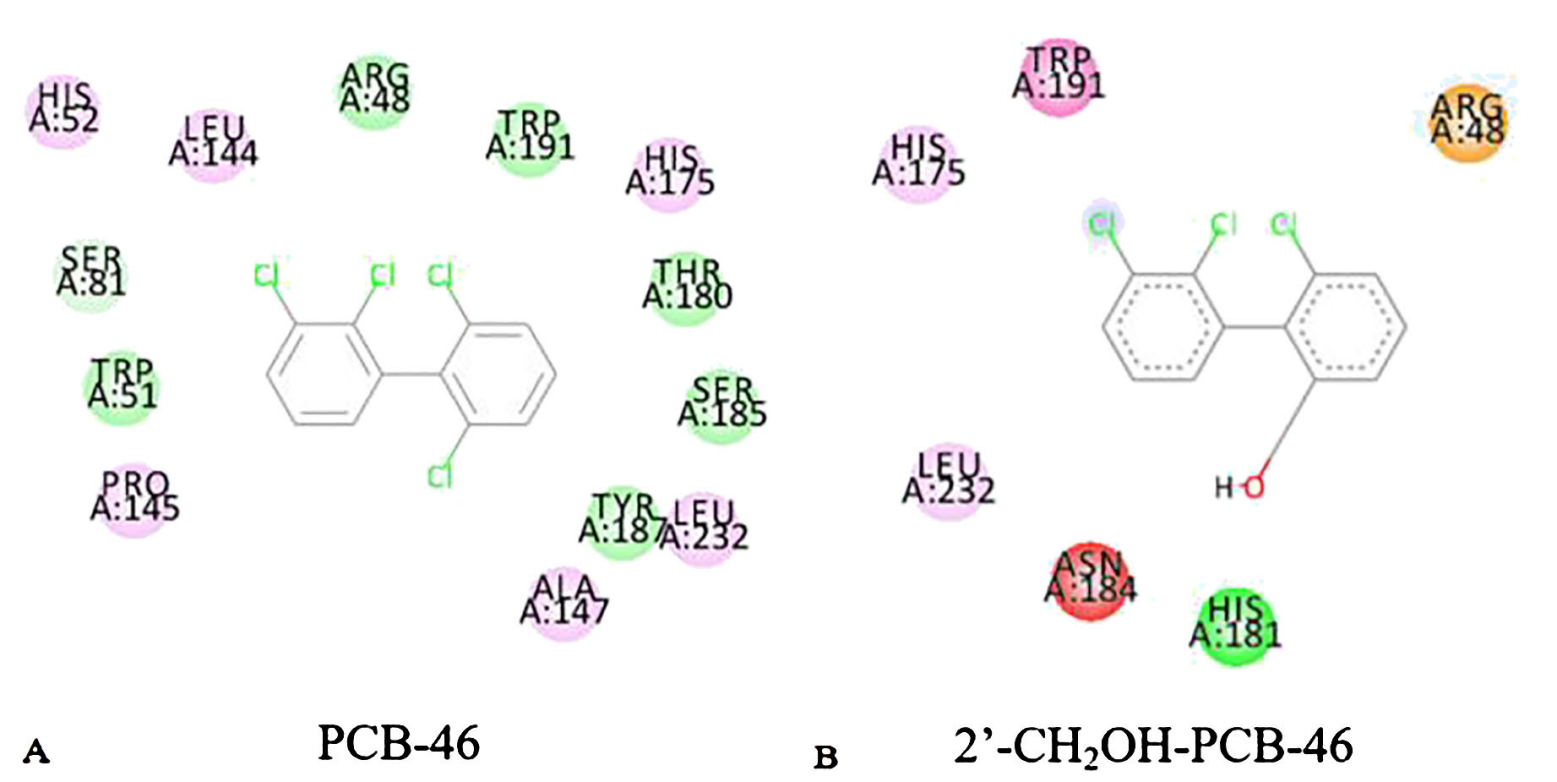
2D diagram of PCB-46 **(A)** and 2′-CH_2_OH-PCB-46 **(B)** binding to plant-degrading enzymes.

Van der Waals forces, as attractive forces, become stronger as the distance between the molecule and the protein decreases (Staahlberg et al., 1992). We extracted the situation near the binding site between PCB molecules and plant enzymes, and found that the van der Waals force is the highest. **Figure 3** illustrates that the main force around PCB-46 binding to plant resistance enzymes is van der Waals forces (involving amino acid residues SER81, ARG48, TRP191, THR180, SER185, TYR187, and TRP51), followed by electrostatic forces (involving amino acid residues HIS52, LEU144, HIS175, LEU232, ALA147, and PRO145). After docking, 2′-CH_2_OH-PCB-46 with plant resistance enzymes exhibited peripheral forces, including van der Waals forces (involving amino acid residue HIS181), electrostatic forces (involving amino acid residues HIS175 and LEU232), π–π stacking (involving amino acid residue TPR191), unfavorable interactions (involving amino acid residue ASN184), and π–cation interactions (involving amino acid residue ARG48).

Molecular dynamics methods were used to calculate the binding energies and van der Waals forces of PCB-46 and 2′-CH_2_OH-PCB-46 with plant resistance enzymes. The results showed that PCB-46 had a binding energy of 122.444 kJ/mol and van der Waals force of 149.006 kJ/mol with plant resistance enzymes, while 2′-CH_2_OH-PCB-46 had a binding energy of 110.059 kJ/mol and van der Waals force of 128.887 kJ/mol with plant resistance enzymes. Compared to PCB-46, 2′-CH_2_OH-PCB-46 exhibited a decrease in binding energy and a significant decrease (by 13.50%) in the surrounding van der Waals forces. These results from molecular dynamics calculations further supported the idea that PCB-46 had a greater binding energy due to greater surrounding van der Waals forces, resulting in an opposite effect for 2′-CH_2_OH-PCB-46, where the reduced van der Waals forces led to increased distance between the molecule and the enzyme, resulting in weaker binding and reduced toxicity of 2′-CH_2_OH-PCB-46 on plants.

Furthermore, an analysis of the impact of introducing the -CH_2_OH group on the changes in van der Waals forces of PCB-46 revealed that the molecular volume of 2′-CH_2_OH-PCB-46 increased by 3.84% compared to that of PCB-46, with volumes of 71.95 cm^3^/mol and 69.29 cm^3^/mol, respectively. A larger molecular volume increases the distance between the molecule and the protein, weakening the van der Waals forces and attraction between the protein and the molecule (Roth et al., 1996). Therefore, the introduction of the -CH_2_OH group resulted in an increased molecular volume and weakened van der Waals forces of 2′-CH_2_OH-PCB-46, ultimately improving its plant resistance compared to PCB-46.

**Figure 4:**
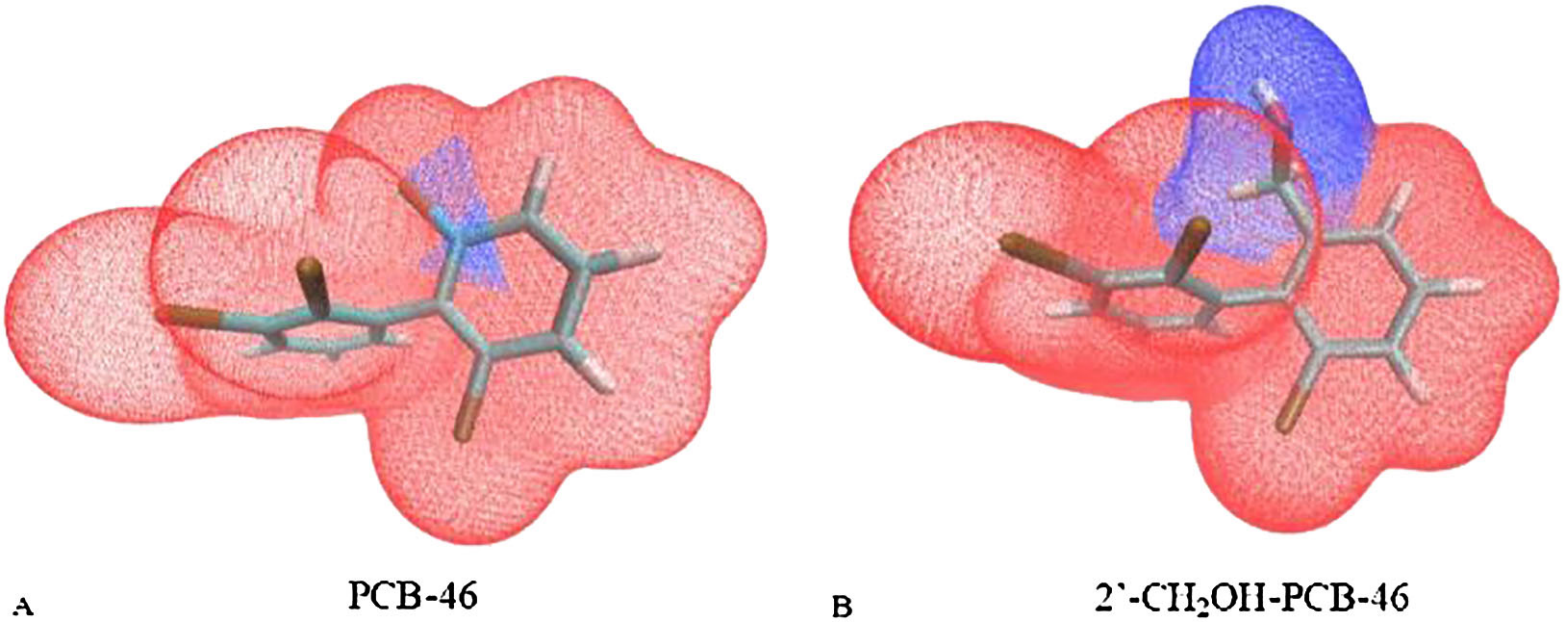
Van der Waals surface area of segments 1 and 2 of PCB-46 **(A)** and 2′-CH2OH-PCB-46 **(B)**.

Additionally, the van der Waals surface area fraction of the virtually modified fragments in PCB-46 and 2′-CH_2_OH-PCB-46 was calculated using the “Quantitative Molecular Surface Analysis” function of Multiwfn software (Qu et al., 2012), with the electron density of the equivalent surface set to 0.002 a.u. In this study, the -Cl substituent region of PCB-46 was designated fragment 1, and the -CH_2_OH substituent region of 2′-CH_2_OH-PCB-46 was designated fragment 2. **Figure 4** illustrates the van der Waals surface effects of PCB-46. The calculations revealed that the van der Waals surface area of fragment 1 (depicted in blue) constituted 1.91% of the total van der Waals surface area of PCB-46, while the van der Waals surface area of fragment 2 (depicted in blue) constituted 17.93% of the total van der Waals surface area of 2′-CH_2_OH-PCB-46. Dispersion forces, an important form of van der Waals forces, exist between all molecules or atoms (Roth et al., 1996). The greater the molecular weight of a molecule is, the greater its deformability, leading to stronger intermolecular dispersion forces (Liu et al., 2019). Compared with fragment 1 of PCB-46, the reduction in the molecular weight of fragment 2 results in a decrease in the dispersion force exhibited by 2′-CH_2_OH-PCB-46. Additionally, fragment 2 occupies a larger portion of the limited total van der Waals surface, further diminishing the dispersion force of 2′-CH_2_OH-PCB-46 and reducing the van der Waals force. The reduction of molecular weight leads to a decrease in the energy of dispersion forces, while the larger molecular volume occupies a greater area within the limited van der Waals surface area, thus resulting in an overall decrease in the van der Waals force. Consequently, when introducing hydrophobic groups, it is important to consider both the molecular weight of the group itself and the alteration in molecular volume following virtual modification. This approach is beneficial for enhancing plant resistance to PCBs.

## 4 Conclusion

A 3D-QSAR pharmacophore model for the combined effects of PCBs on plant stress resistance and degradation was constructed, which can better express the combined pharmacophore characteristics of PCBs on plant stress resistance and degradation. The Hypo 1 pharmacophore model contains one hydrophobic and two aromatic ring features, indicating that these two features have a significant impact on the combined effects of pollutants on plant stress resistance and degradation. Since the aromatic ring feature is a characteristic of PCB structure, adjusting the hydrophobic characteristics of PCBs in plants can further alleviate PCB stress resistance and improve PCB degradation.

Using PCB-46 with the highest comprehensive effect value as a template molecule, molecular virtual modification technology was used to improve the resistance and degradation of PCBs in plants. The mechanism analysis results of the Hypo 1 pharmacophore model were verified, indicating that hydrophobic characteristics affect the resistance and degradation of PCBs in plants, significantly affecting the degradation effect of pollutants in plants.

In-depth analysis of the mechanism changes in plant stress resistance and plant degradation improvement before and after virtual modification of PCBs revealed that hydrophobic groups have a greater impact on plant degradation of pollutants. The improvement in plant degradation is biased toward hydrophobic groups with weaker hydrophobicity compared to chlorine atoms, while the improvement of plant stress resistance tends to be associated with hydrophobic groups with smaller molecular weights compared to chlorine atoms and an increase in the overall molecular volume after virtual modification.

## Data Availability

The original contributions presented in the study are included in the article/supplementary material. Further inquiries can be directed to the corresponding author.
